# Methodological Considerations for Use of Routine Health Information System Data to Evaluate Malaria Program Impact in an Era of Declining Malaria Transmission

**DOI:** 10.4269/ajtmh.16-0734

**Published:** 2017-09-27

**Authors:** Ruth A. Ashton, Adam Bennett, Joshua Yukich, Achuyt Bhattarai, Joseph Keating, Thomas P. Eisele

**Affiliations:** 1 Center for Applied Malaria Research and Evaluation, School of Public Health and Tropical Medicine, Tulane University, New Orleans, Louisiana;; 2 Malaria Elimination Initiative, Global Health Group, University of California San Francisco, San Francisco, California;; 3 President's Malaria Initiative, Malaria Branch, Division of Parasitic Diseases and Malaria, Centers for Disease Control and Prevention, Atlanta, Georgia

## Abstract

Coverage of malaria control interventions is increasing dramatically across endemic countries. Evaluating the impact of malaria control programs and specific interventions on health indicators is essential to enable countries to select the most effective and appropriate combination of tools to accelerate progress or proceed toward malaria elimination. When key malaria interventions have been proven effective under controlled settings, further evaluations of the impact of the intervention using randomized approaches may not be appropriate or ethical. Alternatives to randomized controlled trials are therefore required for rigorous evaluation under conditions of routine program delivery. Routine health management information system (HMIS) data are a potentially rich source of data for impact evaluation, but have been underused in impact evaluation due to concerns over internal validity, completeness, and potential bias in estimates of program or intervention impact. A range of methodologies were identified that have been used for impact evaluations with malaria outcome indicators generated from HMIS data. Methods used to maximize internal validity of HMIS data are presented, together with recommendations on reducing bias in impact estimates. Interrupted time series and dose-response analyses are proposed as the strongest quasi-experimental impact evaluation designs for analysis of malaria outcome indicators from routine HMIS data. Interrupted time series analysis compares the outcome trend and level before and after the introduction of an intervention, set of interventions or program. The dose-response national platform approach explores associations between intervention coverage or program intensity and the outcome at a subnational (district or health facility catchment) level.

## BACKGROUND

With a renewed interest in achieving malaria elimination and funding available from a variety of sources for malaria control, many malaria endemic countries have successfully increased coverage of malaria prevention and control interventions as part of their national strategic plans.^1^ As countries consider approaches to sustain these gains or progress toward elimination, there is a continued need for rigorous evaluation to demonstrate the impact of interventions delivered by national control or elimination programs, and to advocate for continued investment in malaria control and elimination.

Impact evaluations of public health interventions typically attempt to attribute changes in a given health outcome to a particular program, intervention, or set of interventions. Common across impact evaluation recommendations of major donor agencies is the need for a counterfactual, allowing one to measure or estimate the change in outcome indicator had the intervention, set of interventions or program never been implemented. However, there are differences between agencies in the requirements for demonstration of causal attribution of outcome to intervention.^2–4^ Impact evaluations can use a variety of inferences to explore attribution of intervention to outcome.^5^ Although experimental designs using randomization have^4^ been viewed as gold standard in evaluation,^6,7^ these methods may not be ethical or appropriate for evaluation of routine activities involving interventions previously demonstrated to be effective under controlled conditions. Quasi-experimental designs allow evaluation of intervention impact under routine program conditions, demonstrating the plausibility of an intervention being causally linked with an outcome.^5,8^ Considering the complex causal pathways and multiple contributing factors involved in evaluation of public health interventions, as well as the lack of true contemporaneous control groups, plausibility evaluation designs may be more appropriate than probability designs under these circumstances.^6,9^

For the purpose of this paper, we define impact evaluation as the potential contribution of one or more interventions delivered under routine operational conditions to change malaria associated morbidity. Additionally, malaria burden is defined for this paper as either malaria case count or malaria incidence, estimated using routine health management information system (HMIS) data.

Although the HMIS structure has already been established in many countries at either national scale or across malaria-endemic settings to routinely collect and report the number of suspected and laboratory-confirmed malaria cases identified at health facilities, these data have typically been overlooked in favor of population-based cross-sectional surveys as the primary data source for impact evaluations. Objections to use of HMIS data are largely due to potential for measurement error and confounding in malaria burden estimates generated from HMIS data, and concern that these limitations may bias subsequent impact estimates.^10–12^ This concern was particularly valid before the expansion of malaria diagnostic testing that has occurred in the past 5 years; when most HMIS data from high burden countries were based solely on clinical cases without confirmation. Consequently, the majority of evaluations assessing impact of malaria interventions applied under routine conditions by national malaria control programs have used population-based, nationally representative cross-sectional survey data (e.g., Malaria Indicator Survey [MIS]) to assess the association between malaria interventions and malaria outcomes.^13–15^ Unfortunately, these data are intermittently gathered after relatively long periods of 2–3 years, limiting our ability to assess impact on longitudinal trends in indicators of malaria morbidity.

Considering the substantial investments already made in HMIS systems, the underutilization of routine HMIS data has been described as an unacceptable inefficiency in resource constrained countries.^12,16^ HMIS data provide a source of longitudinal data for facilities, enabling estimation of malaria incidence rates over time. Furthermore, widespread adoption of malaria rapid diagnostic tests (RDTs) has increased the availability of parasitologically confirmed malaria case data in HMIS.^1^ Adoption of electronic reporting systems such as the District Health Information System 2 (DHIS2) has been shown to improve completeness and timeliness of routine data reporting.^17^ HMIS data have particular value for impact evaluation in low transmission settings, where periodic large-scale cross-sectional surveys may have inadequate power to assess trends in malaria outcomes over time, especially at subnational levels.^18^ Amending these cross-sectional survey designs to have adequate power for such national and subnational examination of trends in low transmission areas would require a large increase in sample size and cost.

The Global Technical Strategy for Malaria 2016–2030 has emphasized the importance of high-quality routine HMIS data by redefining surveillance as a core intervention in malaria control and elimination.^19^ It is therefore likely that the pace of improvements in HMIS data completeness, quality, and use will accelerate in line with the new global strategy. As a result of increasing access to parasitologically confirmed, complete, and representative HMIS data, it is timely to reconsider the applications of these data in impact evaluation.

In this study, we review the literature to describe malaria impact evaluation designs where the primary outcome of malaria burden was generated using routine HMIS data. Limitations and advantages of the various methodologies used are discussed. Cross-cutting methodological issues such as bias and confounding are discussed in relation to generation of outcome estimates from HMIS data, as well as in generation of impact estimates using various analysis approaches.

## METHODS

### Literature search method.

The literature was searched to identify malaria impact evaluations that used routine health information system data. Although not systematically reviewed, the papers were used to describe the range of methodologies used to assess trends in malaria morbidity or impact of interventions and programs on malaria morbidity, when the primary outcome was generated from routine HMIS data. In addition, the approaches used in the literature to address bias in outcome indicators and bias in impact estimates were investigated, with additional recommendations and examples drawn from the wider literature. A selection of malaria impact evaluations using HMIS data are presented in Table 1, to illustrate the range of methodologies identified in the literature. Since only a minority of malaria deaths occur in health facilities, use of HMIS inpatient data to assess trends in malaria mortality may be biased,^20^ unless it can be demonstrated that inpatient mortality trends are representative of mortality trends in the population. As such, HMIS-derived indicators of malaria mortality are not considered in this study.

**Table 1:** Papers identified during literature search presenting malaria trends or impact evaluations using routine HMIS data

Reference	Data extent	Analysis approach	Contextual factors included in analysis interpretation
**Pre–post comparison of rates**			
			
Chanda and others 2012^29^	30 Districts, 2 years’ data. Confirmed malaria: severe and deaths among < 5 seconds, case fatality rate	Pre–post comparison. Logistic regression model including population and % intervention coverage to account for between district differences.	Change in first-line treatment, consequent changes in treatment seeking
Comfort and others 2014^25^	Two hospitals, 6 years’ data. Confirmed malaria outpatient cases and inpatient admissions	Pre–post comparison. Descriptive comparison of mean outcome level pre–post intervention.	All-cause outpatient and inpatient numbers
Louis and others 2015^96^	4 Years’ data from one district. Clinical malaria	Pre–post comparison. Descriptive comparison of outcome level pre–post intervention.	Nonmalaria illnesses diagnosed at health facilities, health-seeking behavior, reporting completeness, service coverage and quality, community-based health insurance scheme, rainfall
Maes and others 2014^97^	Data from one district, over two separate 3 year periods. Confirmed outpatient malaria and malaria admissions	Pre–post comparison. Descriptive comparison of outcome level pre–post intervention.	Temperature, rainfall, flooding, malnutrition, rift valley fever, IRS campaigns and LLIN distribution, larval source management
Masaninga and others 2013^98^	11 Years’ data, national scale. Confirmed malaria admissions and deaths	Pre–post comparison. Descriptive comparison of outcome level pre–post intervention.	Increased funding for malaria control, LLIN and IRS campaigns
Sarrassat and others 2008^30^	5 Years’ data, one facility. Clinical and confirmed malaria.	Pre–post comparison. Descriptive comparison of outcome level pre–post intervention.	Rainfall (qualitative), facility attendance, change from presumptive treatment to confirmatory diagnosis, population movement
Thang and others 2009^99^	3 Years’ data from two districts. Clinical and confirmed malaria	Pre–post comparison. Incidence rate ratios from Poisson regression to describe change in outcome. No covariates	Rainfall, village health worker program
Willey and others 2011^67^	2 Years’ data from 11 health facilities. Confirmed malaria	Poisson regression comparing incidence between intervention and comparison areas, rather than pre–post intervention.	Vaccination coverage, microscopy quality assurance
Yapabandara and others 2015^100^	7 Years’ data, national scale. Clinical and confirmed malaria	Pre–post comparison. Descriptive comparison of outcome level pre–post intervention.	Health system strengthening, targeting of interventions according to microstratification, change in first-line treatment, adoption of RDTs, IRS chemical.
**Descriptive analysis of trends**			
			
Alba and others 2011^36^	4 Years’ data from 14 health facilities. Clinical malaria.	Poisson regression models with village-level random effects to describe trends in outcome over time. No other potential confounders included in model.	Change in first-line treatment and estimated vector control coverage. Internal and external validity of data assessed.
Bhattarai and others 2007^81^	7 Years’ data from 13 facilities, clinical malaria and malaria admissions.	Descriptive analysis of indicators over time and Pearson correlation coefficients assessing linear relationship between rainfall and outcomes.	Speed of ACT rollout, climate, vector control interventions
Ceesay and others 2008^34^	Five health facilities, 7–9 years’ data. Slide positivity, proportion of admissions, and deaths due to malaria.	Trends in outcome tested using χ^2^, and linear regression (without covariates) of log-transformed case count.	Decreasing sales of antimalarial medicines at pharmacies, change in first-line antimalarial. Rainfall data. Changes in socioeconomics, communications, access to education.
Dhimal and others 2014^35^	50 Years’ national data, additional analysis for district-level data from most recent 9 years. Confirmed malaria	Descriptive analysis of trend over period of intervention scale up using linear regression of log-transformed case count. Tested for temporal autocorrelation. No covariates.	Trends in climate variables assessed, not included in models. Changes in vector ecology, insecticide resistance, change in first-line treatment, community passive detection posts, population movement
		Additional pre–post intervention descriptive comparisons.	
Kamuliwo and others 2013^37^	6 Years’ data, national scale. Clinical malaria throughout, confirmed malaria for final 3 years only	Poisson regression models with case count and intervention coverage level (categorised), with district-level random effect. No covariates.	Insecticide resistance, population movement.
Konchom and others 2003^101^	11 Years’ data from 30 border-area districts. Annual parasite incidence, ratio of *Plasmodium falciparum* to *Plasmodium vivax*	Simple descriptive analysis of indicator levels. *t* Tests comparing incidence in border and nonborder districts over time.	Population movement, change in first-line treatment
Maude and others 2014^33^	10 Years’ national data from facilities and community-level. Clinical and confirmed malaria.	Simple descriptive analysis of indicator levels and correlations.	Change in RDT, expansion of village malaria worker program, vector control, migration, deforestation
Mufunda and others 2007^102^	6 Years’ data, national scale. Clinical malaria	Simple descriptive analysis of indicator levels. Linear regression to explore association interventions with outcome.	Change in first-line treatment
Mukonka and others 2014^103^	7 Years’ data, from 11 facilities in one district. Clinical and confirmed malaria.	Simple descriptive analysis of indicator levels over time	Insecticide resistance, population movement, changes in access to care and case reporting
Ngomane and others 2006^38^	9 Years’ data from one district. Confirmed malaria, from passive and active surveillance. Malaria mortality.	Descriptive analysis of indicator levels and χ^2^ test for trend over time. ARIMA model fitted to assess effect of climate on outcome, accounting for temporal autocorrelation.	Changes in first-line treatment, population movement, behavioral factors in some age groups, agricultural practices
Nyarango and others 2006^39^	5 Years’ data, national level. Clinical and confirmed malaria	ARIMA model testing association between interventions and outcome, accounting for temporal autocorrelation. Rainfall included in model as covariate.	Change in first-line treatment, diagnostic quality assurance, health-seeking behavior, quality of case management
Okiro and others 2007^40^	8 Years’ data from hospitals. Confirmed malaria inpatient admissions	Seasonally adjusted linear regression models to examine trends. Models accounted for temporal autocorrelation. Rainfall, nonmalaria admissions and seasonality included as covariates.	Per capital ITN distribution, change in first-line treatment
Okiro and others 2011^41^	10 Years’ data from five hospitals. Confirmed malaria inpatient admissions.	ARMAX models to examine long-term trends in outcome. Models accounted for temporal autocorrelation, rainfall and nonmalaria admissions included as covariates.	Changing diagnostic practices, abolition of user fees, differences in access to effective treatment between districts
Okiro and others 2013^42^	11 Years’ data from four hospitals. Suspected malaria inpatient admissions	ARMAX models to examine long-term trends in outcome. Models accounted for temporal autocorrelation. Rainfall and nonmalaria admissions included as covariates.	ITN distribution timeline, change in first-line treatment, adherence to national treatment guidelines
Otten and others 2009^26^	7 Years data from 13 facilities in one country, 7 years’ data from 19 facilities in a second country. Outpatient confirmed malaria, inpatient malaria admissions.	Linear regression on case count data to describe trend over time.	Nonmalaria health facility attendance, timing of LLIN distributions, introduction of health insurance schemes, civil conflict resolution
		Additional pre–post intervention descriptive comparisons.	
**Interrupted time series**			
			
Aregawi and others 2011^47^	4 Years’ pre- and 1 year post-intervention data, six inpatient facilities. Confirmed malaria outpatients and admissions, malaria inpatient deaths.	Interrupted time series using log-linear regression model. No potential confounders included in model.	Climate, urbanization, and socioeconomic development
Aregawi and others 2014^48^	5 Years’ pre- and 6 years’ post-intervention data, outpatient and inpatient, from 41 hospitals. Clinical and confirmed outpatient malaria, confirmed malaria admissions and deaths.	Interrupted time series using ARIMA model, accounting for temporal autocorrelation. No potential confounders included in model.	Linear association examined between rainfall and case count and slide positivity. Rainfall compared between pre–post intervention periods. Nonmalaria OPD and IPD data discussed
Bukirwa and others 2009^51^	One health center, 8 months pre- and 16 months post-intervention data. Clinical malaria and slide positivity rate	Interrupted time series using ARIMA model, accounting for temporal autocorrelation. Covariates included age sex, rainfall.	Changes in proportion of suspected malaria cases sent for diagnostic test, ACT scale-up, ITN distribution interventions
Karema and others 2012^46^	11 Years’ data from 30 hospitals	Interrupted time series using log-linear regression model. No potential confounders included in model, but accounts for temporal autocorrelation.	Rainfall and temperature trends, laboratory quality assurance, initial targeting of ITNs to children under five
Kigozi and others 2012^49^	5 Years’ data, one health facility. Slide positivity rate	Interrupted time series, correcting for temporal autocorrelation. Include age and monthly seasonality as covariates	Change in insecticide used for IRS, changes in ITN coverage and use
Landoh and others 2012^50^	6 Years’ data, national scale. Clinical and confirmed malaria	Interrupted time series using ARIMA model, accounting for temporal autocorrelation. Includes rainfall and temperature as covariates.	Increased access to health care, roll out of RDTs.
Santelli and others 2012^52^	5 Years’ data from three districts. Confirmed malaria (active and passive surveillance)	Interrupted time series using log-linear regression model, including seasonality and month-intervention interaction term.	Epidemic prior to intervention, local malaria control management, vector control, reduced efficacy of standard treatment course
Teklehaimanot and others 2009^53^	7 Years’ data, national scale. Confirmed malaria, malaria admissions and deaths	Interrupted time series using spline regression model, including seasonality as covariate. Separate time series model assessing change in rainfall.	Health service coverage and access
**Dose-response analysis**			
			
Bennett and others 2014^24^	3 Years’ routine HMIS data, national scale (1,693 facilities). Confirmed malaria.	National platform approach (district-level dose-response). Covariates included treatment seeking, climate, health care access, testing rate, and reporting rate	Regional population movement, insecticide resistance, potential endogenous relationships between outcome and explanatory variables
Graves and others 2008^54^	6 Years’ data, national scale. Clinical malaria.	National platform approach (district-level dose-response), accounting for temporal correlation. Covariates included rainfall and vegetation cover (NDVI) indicator	Change in first-line treatment, presumptive treatment during high transmission season in one district

ACT = artemisinin-based combination therapy; ARIMA = autoregressive integrated moving average; ARMAX = autoregressive moving average model with exogenous inputs; HMIS = health management information system; IPD = inpatient department; OPD = outpatient department; RDT = rapid diagnostic test.

## HEALTH MANAGEMENT INFORMATION SYSTEM

The core function of an HMIS is to collect, transmit, and analyze indicators required for health system management.^21^ The current review focusses on health facility-based HMIS data, but is also relevant to any parallel malaria-specific reporting systems operating in countries. In general, clinical data from individual patients are aggregated by age category (< 5 or ≥ 5 years) at facility-level each month, then reported to the supervising administrative unit (e.g., district) using a standardized format. Districts review and analyze data received from facilities, provide feedback, and may aggregate data into district totals to submit to the next level (e.g., region).

Some countries have begun reporting malaria diagnoses made by community health workers using RDTs, either to HMIS or through parallel reporting structures.^22^ HMIS usually does not include private health facility data, but there is growing interest in the utility of private sector data in malaria surveillance, particularly in elimination settings.^23^ Following expansions in mobile telephone network coverage and reducing cost of mobile internet, some countries are beginning to transition their HMIS systems from paper- to cloud-based systems, such as the DHIS2.^17,24^

HMIS indicators particularly relevant to malaria impact evaluation include all-cause outpatient attendance, clinical malaria diagnoses, number receiving parasitological test (either by microscopy or RDT), test positivity rate, diagnostically confirmed malaria cases, and malaria inpatient admissions. Monthly malaria case count or monthly malaria incidence per 1,000 catchment area population are the main outcome indicators for impact evaluations included in this review.

## IMPACT EVALUATION DESIGNS USING HMIS DATA

### Pre–post intervention comparison.

The simplest approach to use of HMIS data in impact evaluation is through pre–post ecological comparison, where malaria case count or incidence is estimated before and after the intervention and tested statistically for evidence of change.^25^ However, malaria transmission is influenced by a range of factors (e.g., climate and environment) and the main limitation of pre–post comparison designs is an erroneous assumption that all changes in malaria case rate over time are attributable to the intervention.^26,27^ These simple pre–post comparisons are consequently vulnerable to confounding from secular trends and other contextual changes over time.^28^

Pre–post comparison studies can be improved by describing advances or declines in relevant contextual factors and potential confounders or effect modifiers, and presenting a balanced interpretation of impact estimates considering the limitations of available data and potential for bias in impact estimate.^29,30^

When appropriate nonintervention areas (here termed “contrast” areas since they are not true controls) can be either observed or modeled, the difference in difference (DiD) estimator can be applied; this estimates the difference in pre–post estimators between intervention and contrast areas. The DiD approach therefore enables pre–post comparison designs to take into account underlying trends in outcome level over the intervention period. To avoid biased impact estimations, DiD requires a valid contrast area or counterfactual, representing the change in outcomes that would have been experienced by the treatment group in the absence of interventions.^31^ In practice, DiD estimators can be generated through use of multivariate logistic regression models including dummy variables identifying intervention/contrast group and pre/post intervention period, together with an interaction term of intervention/contrast group and pre/post intervention period. DiD is most commonly used for population-based survey data.^32^

### Descriptive analyses of trend.

Descriptive analysis of indicators over time can be a useful component of an impact evaluation, providing quantitative or qualitative interpretation of change over time in outcome indicators. Simple descriptive interpretation analysis of HMIS data can be strengthened by presenting plots of multiple indicators over time, conducting analysis at both national and subnational level, and description of contextual changes which may be influencing observed trends.^33^ Descriptive analyses of HMIS trends can be supplemented by χ^2^ or *t* tests for trend, as well as linear regression of log-transformed case count data.^34^

Logistic regression approaches enable examination of trends in outcome indicators after accounting for contextual factors that varied over the evaluation period and may have confounded or interacted with associations between exposure and outcome. Linear regression of case count data should be avoided, since this could result in prediction of negative case counts.^26,27^ Multiple examples exist using linear regression of log-transformed case count data,^34,35^ or Poisson or negative binomial regression of case count data.^36,37^ Autoregressive integrated moving average (ARIMA) and other time-series model structures are appropriate for analysis of trends in HMIS indicators over time, since they account for temporal autocorrelation and allow adjustments for seasonal trends.^38–42^

Although considering contextual factors in analysis interpretation does strengthen pre–post comparisons, this approach continues to be limited by the lack of counterfactual for describing the change in malaria outcome over the evaluation period in the absence of an intervention.

### Interrupted time series.

Interrupted time series (ITS) analysis involves comparison of level and mean trend in outcome indicators before and after a breakpoint.^43,44^ Although more than one breakpoint can be considered in ITS models, the approach is most suited to single interventions that are rolled out over short periods with consistent intensity. In settings where interventions required more time to achieve full implementation (e.g., staggered long-lasting insecticide-treated net (LLIN) distributions across a country over a period of 6 months) after introduction, or where a lag is expected between introduction of the intervention and effect on outcome, ITS models can be adapted to test for the optimal breakpoint, or divided into preintervention, intervention rollout, and postintervention segments. However, this weakens the ITS approach since testing numerous breakpoints or having a long scale-up period increases the plausibility of changes in outcome being attributable to other potentially confounding factors.^45^

Some ITS analyses simply examined associations between timing of intervention scale-up and change in case incidence^46,47^; however, it is recommended that contextual factors are included in ITS models to avoid erroneous attribution of changes in outcome to the intervention. Climate data are commonly included as covariates in ITS analyses, both for evaluations comprising a small number of facilities and for national level analyses.^48–50^ Many ITS models use ARIMA structures,^48,50,51^ but other time series regression models can be used.^46,52,53^ Regardless of modeling structure, ITS should incorporate terms to address temporal autocorrelation, and seasonality effects should be modeled and removed from a time series before assessing treatment impact.^45^

A further extension is to analyze a contrast group time series using ITS, to explore if other contextual changes occurred which may confound any observed association between intervention breakpoint and change in malaria burden indicators. For example, ITS could be applied to all-cause or nonmalaria outpatient visits, to investigate if there were changes in facility attendance over the evaluation period. Although not strictly a counterfactual scenario, examination of trends in other indicators available from HMIS can give insight to potential confounding or contextual changes occurring at the same time as the intervention.

### Subnational dose response.

The national platform approach to impact evaluation explores a dose-response relationship between intervention and outcome at a subnational (e.g., district) level.^9,32^ The method overcomes challenges in identifying a valid counterfactual in environments of universal scale-up, or where ethical concerns preclude withholding of interventions. Continuous monitoring of different levels of contextual indicators, and collection of additional data before, during and after the intervention are recommended for the national platform approach, together with use of multiple analytical techniques to address potential biases in the data.^9^

Key to this dose-response analysis approach is the availability of intervention and covariate data at the subnational level. Intervention data may be available in district health authorities’ records, such as number of households receiving indoor residual spray, or total LLINs distributed per capita. Alternatively, district-level data on intervention coverage can be extracted from population surveys such as the DHS or MIS.

Examples of use of the national platform approach for impact evaluation are available from Eritrea and Zambia.^24,54^ Both evaluations used routinely collected HMIS data aggregated at the district-level to conduct a national evaluation of changes in malaria burden over a period of malaria control intervention scale-up. The Eritrea evaluation used program records of vector control coverage (including decay functions to represent LLIN losses and reduced effectiveness of indoor residual spray (IRS) insecticide over time) together with climate data, to assess the dose-response relationship between intervention and malaria case count.^54^ The Zambian study used the same principles, but with a more complex approach to generation of complete outcome and intervention data; a Bayesian framework was used incorporating temporal and spatial autocorrelation, and a range of contextual factors considered in the model.^24^

## FURTHER CONSIDERATIONS FOR IMPACT EVALUATION DESIGN USING HMIS DATA

### Overcoming common sources of bias in HMIS data to generate outcome indicators.

HMIS data have been underutilized in impact evaluation due to concerns that malaria burden estimates generated from HMIS data are very prone to bias. Most sources of bias in case incidence data would only be expected to lead to a biased estimate of intervention or program impact if the factor was associated with the intervention under evaluation (as classically defined for confounding variable). The exception is nondifferential misclassification bias, which would be expected to lead to an underestimate of intervention or program impact. Nevertheless, this section discusses methods to minimize bias when generating outcome indicators used for impact evaluation from routine HMIS indicators.

Bias in estimates of malaria case incidence from HMIS data can result from a range of factors: challenges in estimating size of catchment populations, changes in treatment-seeking, variable access to and use of parasitological diagnosis, and incomplete recording and reporting of data (both missing monthly reports and incomplete registration of patients in registers).

Defining the catchment population for a health facility enables standardized estimates of case incidence to be used as the primary outcome measure in impact evaluation. Case counts are influenced by the size and composition of the population being served by the health facility; facilities serving larger populations are expected to have more cases than those serving smaller populations. However, catchment populations may not be static over time, and individuals may choose not to attend their closest health facility,^55^ creating challenges in appropriate estimation of catchment populations. Approaches used to estimate catchment population include generating Euclidean buffers around facilities,^41^ Thiessen polygons,^42^ estimating travel time using road networks, land use and altitude gradients,^24,56,57^ a variable market approach,^58^ the Huff competitive market model,^59,60^ or calculation of cumulative case ratios.^61^ Where population data are available by age (categorical or continuous), malaria incidence rates can be disaggregated. This is particularly recommended where age structures may vary over time or space, and can also be valuable in tracing changes in transmission intensity indicated by peak shift of symptomatic and severe malaria cases.^62^ If no population data are available within the country, district-level population can be extracted from global geo-referenced population databases,^63^ and analysis conducted at district-level using aggregate HMIS indicators.

In settings where not all who fall ill seek treatment, and not all seeking care go to a public health facility, HMIS data will systematically underestimate malaria case rates for the given community. Less commonly, where variations in health-seeking behavior are associated with the intervention of interest, this may result in biased estimates of intervention impact. Estimates of health-seeking behavior can be extracted and modeled from MIS or DHS data, where women are asked if and where they took their children for medical treatment if they were recently unwell.^24^ Questions in these surveys can also be used to estimate the proportion of the population who seek treatment from the private sector, and are consequently underrepresented in routine HMIS data. A simple alternative is include nonmalaria outpatient attendance at the facility to adjust for changes in all-cause health-seeking behavior over the evaluation period, assuming that there have been no changes in total catchment population over the evaluation period.^40^

Where HMIS data report only clinical (not parasitologically confirmed) malaria, symptom-based diagnostic algorithms are known to have low specificity.^64^ In the absence of confirmatory testing, a health worker’s decision of whether to diagnose malaria may be influenced by factors related to the health worker (e.g., level of training, workload) or the patient characteristics (e.g., age, treatment expectation), or by broader contextual factors.^65^ Accounting for low specificity of clinical diagnosis in analysis is very challenging; it is recommended that if possible, only confirmed malaria cases be used to derive primary outcome indicators for impact evaluations. Fortunately, widespread scale-up of RDTs has increased the likelihood of diagnostic confirmation among suspected malaria cases, making data on confirmed malaria cases increasingly available. However, changes in access to parasitological diagnosis have the potential to bias assessment of temporal trends in confirmed malaria rates, unless malaria testing rates among febrile patients, and periods of diagnostic tool stock-out are incorporated into analysis.^66^ Settings where RDTs were initially used for diagnosis in children under five, then subsequently expanded to all ages have additional complexity, since both testing rates and test positivity rates during the under-five targeted RDT period may be biased by children’s increased risk of clinical malaria episodes compared with older age groups. Use of test positivity rate as an outcome indicator is not advised for impact evaluations, since this indicator may be biased by a wide range of factors, including the type of diagnostic tool, availability of diagnostics, workload at the facility, season, and patient characteristics. Programmatic changes such as adoption of RDTs at facilities, stock-outs, changes in RDT brand or antigen combination, introduction of systematic quality assurance and quality control approaches for monitoring RDT users, or even timing of refresher training for health workers on malaria case management are additional contextual factors which could be incorporated into impact evaluation.^67^

Incomplete reporting of data to the HMIS leads to systematic underestimation of disease rates. The reporting completeness of any HMIS, together with many other surveillance system attributes, should be carefully evaluated on some periodic basis.^68^ Where the level of underreporting is associated with the intervention under evaluation, impact estimates may be biased unless underreporting is addressed in analysis. Incomplete reporting may result from inconsistent submission of monthly reports by facilities, omission of specific indicators within submitted reports, or patient data being only partially recorded in outpatient and laboratory registers. To address missing reports or indicators, imputation methods can be used if relatively few data are missing from a time series.^69^ Models with spatial and temporal correlation structures may also inform estimation of missing data, weighting estimates according to known values which are close in space and time.^70^ Alternatively, estimates of reporting completeness can be included as a variable in analysis; a preferred method over direct adjustment of imperfect incidence data, particularly at small temporal and spatial scales.^71^ Underreporting due to patients not being recorded in registers is substantially more challenging to address in analysis, and may require restriction of analysis to sentinel facilities or those known to have highly complete data.^34,54^ However, restricting impact evaluations to the best-performing facilities has potential to bias impact estimates if intervention quality, coverage or use in these populations differs to that among populations served by underperforming facilities which are excluded from analysis. When possible, HMIS data from all health facilities should be included in impact evaluations.

### The counterfactual.

Presenting an appropriate counterfactual scenario is recommended by multiple major donor agencies for impact evaluations.^2–4^ However, appropriate counterfactuals can be very challenging to identify under operational contexts in most malaria-endemic countries.

When using a pre–post comparison design, the counterfactual assumes that no change would have occurred in the outcome without the intervention. A crude approach to estimate a counterfactual in pre–post evaluations is to compare changes in nonmalaria HMIS indicators, such as all-cause outpatient attendance or mean rates of another disease, before and after the intervention. However, this approach should be used with caution, since other the disease used as a counterfactual could have experienced a natural epidemic, or been subject to other interventions not directly considered in the evaluation.

It may be possible in some settings to use a comparator area as a counterfactual, where the assumption is that had the intervention has not been applied, trends in the outcome indicator would be the same in the intervention and comparator areas. This approach may be useful where interventions were targeted to specific areas, or rolled out as part of stepped-wedge trial designs.^32,66,72^

The counterfactual for most ITS approaches assumes that the mean trend for the preintervention period would have continued in the absence of the intervention. If a time series model was fitted to preintervention period data including climate and other temporally dynamic covariate data, the regression model could be extended to predict a counterfactual for the postintervention period using climate covariates for that period. Generating counterfactuals using ITS and regression extrapolation is challenging in environments with limited preintervention data due to challenges in fitting preintervention models, and where recent systematic changes in diagnosis or data collection bias preintervention incidence estimates.

Dose-response evaluation designs make use of subnational variations in intervention coverage or program intensity to estimate impact on the malaria morbidity outcome indicator. In these evaluation designs, a counterfactual may be represented by a “zero-dose” district, but are not explicitly required in analysis.

### Contextual and confounding factors.

When designing an evaluation of interventions applied in complex operating environments, it is recommended to include data on relevant contextual factors and their variation over time.^73,74^ In this paper, we have referred to contextual factors as a broad group of qualitative or quantitative factors that may potentially bias exposure, outcome, or impact estimates. We consider confounding factors and effect modifiers to be specific contextual factors with precise epidemiological definitions: confounders being variables that are both associated with exposure and outcome but not on the causal pathway, while effect modifiers are alter the effect of a putative causal factor being investigated.^75^ The Bradford Hill considerations can assist in describing the strength of evidence for causal associations in quasi-experimental impact evaluation designs.^76,77^ Logic models and theoretical frameworks are a useful approach to describe hypothesized causal pathways, contextual factors, confounders and effect modifiers, informing evaluation design, and interpretation in complex settings.^78–80^

Potentially relevant contextual factors for malaria impact evaluations include other health programs being implemented that may influence malaria outcomes (e.g., vaccination, deworming, nutrition programs), climatic conditions (specifically temperature, precipitation and relative humidity, or proxy measures such as vegetation indices), and any other events in the community (e.g., food insecurity, population movements, economic shocks, or political events) that occurred over the period of observation and which have the potential to alter any of the indicators being monitored. A number of studies have used a mixed approach to evaluation, incorporating some contextual factors directly into models as potential confounders or effect modifiers and considering additional contextual factors in models interpretation as part of a plausibility approach.^81–83^

Indicators such as access to health services, treatment-seeking behavior, and reporting completeness should be examined for association with the intervention under evaluation, and ideally be incorporated into analysis to strengthen internal validity of HMIS data. In settings with mobile or migratory populations, indicators of human population movement may be valuable to incorporate in large-scale evaluations, since changes in malaria susceptibility and risk of transmission may occur following population movements.^84,85^

Environmental variables are recommended for inclusion in time series models, since vector populations and therefore malaria transmission intensity are influenced by climatic conditions.^86^ Failure to incorporate environmental covariates in models may lead to erroneous attribution of impact to interventions, if malaria outcomes are (partially or fully) due to a change in climatic conditions rather than the intervention. Although some small-scale impact evaluations have used local weather station data, the use of satellite-derived environmental data is becoming increasingly popular.^24,38,40,50^ A systematic audit of an extensive library of environmental covariates led to development of a set of covariates which were found to reliably model malaria risk *Plasmodium falciparum* parasite rate (*Pf*PR_2–10_) patterns and how these patterns change through time.^87^ However, anomalies in rainfall, temperature, and vegetation indices are the most widely used environmental covariates in malaria models. Many climate variables are best considered as lagged variables, due to the time required for a change in climate to manifest as a change in a malaria outcome.

Where evaluations incorporate data from a range of malaria transmission settings, an estimate of transmission intensity such as modeled *Pf*PR_2–10_ can be incorporated in analysis as a potential effect modifier.^15,88^ Maps of modeled *Pf*PR_2–10_ are publically available at 5-km resolution,^89,90^ enabling extraction of *Pf*PR_2–10_ estimates for specific sites.^91,92^ However, since *Pf*PR_2–10_ estimates are generated using models that include environmental variables, interventions, seasonality and other confounders, including *Pf*PR_2–10_ in impact estimation models may bias effect estimates upward due to circularity.

Considering the range of contextual factors, confounders and effect modifiers which could be included in impact evaluations, theoretical frameworks are recommended to explore the hypothesized relationships between each factor and the exposure and outcome indicators, and begin prioritizing factors on which to gather data and incorporate in analysis. In malaria impact evaluations utilizing HMIS data, priority contextual factors include indicators of access to health care and treatment seeking for malaria, malaria diagnostic practices, and climate data. In many settings, indicators of access to health care and treatment seeking are available in periodic national surveys such as DHS, while diagnostic practices will be documented or known by national malaria program staff. Satellite-derived climate data are readily available for many countries from sources such as the U.S. Geological Society.

### Autocorrelation.

Time series data are typically autocorrelated, meaning that an observation may be correlated (positively or negatively) with observations one or more period lags prior.^45^ Failure to account for autocorrelation in analysis of time series can result in erroneous findings of statistical significance.^44,93^ Similarly, due to the correlated nature of data across spatial units, spatial autocorrelation should be considered in analysis of HMIS data from large numbers of facilities with known locations. Use of methods such as ARIMA automatically adjusts for temporal autocorrelation.^94^ A simpler approach is to incorporate a lagged outcome variable in logistic regression models to account for temporal correlation.^54^ The lag may be 1 month or more, according to the autoregressive structure of the data.

Accounting for autocorrelation is also important for standard error and 95% confidence interval estimation in models assessing the significance of exposure variables on the outcome of HMIS-derived rates. Although missing data can be generated in models using spatial and temporal interpolation methods,^70^ using a Bayesian framework for analysis allows uncertainty in outcome data and other indicators to be propagated forward through models into impact estimates.^24,95^

## CONCLUSIONS

HMIS data are common across all malaria-endemic countries, yet have been underused in impact evaluation due to quality and bias concerns. Increasing investments in malaria surveillance, broad availability of RDTs, improved systems to record, transmit, and process these results, and the reduced power of population malariometric surveys in areas of low transmission prompt a necessary reconsideration of the utility of HMIS data in impact evaluation.

The impact evaluations reviewed fall into four main designs: pre–post intervention studies, descriptive analyses, ITS, and dose-response analyses. Pre–post designs and descriptive analysis are limited by a lack of counterfactual, but can be bolstered by presenting a theoretical framework of contextual, confounding, and interacting factors which could bias impact estimation. ITS and dose-response designs are more rigorous approaches to impact estimation using HMIS data. ITS is appropriate when interventions have been scaled up nationally and rapidly, relevant contextual variables are included in models, and sufficient preintervention data are available to generate a counterfactual. Where subnational data are available describing intervention coverage or intensity over time, a dose-response analysis strategy incorporating relevant subnational data on potential confounders is appropriate. The methods discussed for analysis of HMIS data in impact evaluation range from simple to complex, yet most can present meaningful information if presented using a plausibility approach.^5,6^

Multiple approaches have been presented to address some of the common biases in HMIS data, including methods to estimate the catchment population of facilities, incorporate estimates of health service access and health-seeking behavior, diagnostic test use and validity, as well as incomplete reporting. These variables should be either directly included in models or explored in result interpretation along with other contextual factors, considering if, and to what extent, these may have influenced the observed data or estimated impact. We recommend that confirmed rather than clinical malaria be used as the primary outcome indicator when using HMIS data in impact evaluation, due to low specificity of symptom-based diagnostic algorithms for malaria. A population denominator should also be included, either by use of malaria incidence as the primary outcome, or inclusion of health facility catchment populations in Poisson models using case count data.

As malaria programs continue to strengthen and expand to pursue malaria elimination, historical and prospective use of HMIS data for rigorous impact evaluations will be needed. Improvements in health system performance, access to health care, increasing availability of RDTs for parasitological confirmation of suspected malaria cases, combined with advances in electronic information systems (data capture and transmittal) will increase the validity of indicators derived from HMIS making these data increasingly useful and efficient for rigorous impact evaluations.

## References

[1] World Health Organization, 2015 *World Malaria Report 2015* Geneva, Switzerland: WHO. Available at: http://apps.who.int/iris/bitstream/10665/200018/1/9789241565158_eng.pdf?ua=1. Accessed December 15, 2015.

[2] United States Agency for International Development, 2011 *USAID Evaluation Policy* Available at: https://www.usaid.gov/evaluation/policy. Accessed May 18, 2016.

[3] The World Bank, 2016 *Poverty Reduction and Equity: Overview of Impact Evaluation* Available at: http://web.worldbank.org/WBSITE/EXTERNAL/TOPICS/EXTPOVERTY/EXTISPMA/0,,menuPK:384339∼pagePK:162100∼piPK:159310∼theSitePK:384329,00.html#whatis. Accessed May 18, 2016.

[4] UK Department for International Development, 2013 *International Development Evaluation Policy* Available at: https://www.gov.uk/government/publications/dfid-evaluation-policy-2013. Accessed May 18, 2016.

[5] Habicht JP Victora CG Vaughan JP, 1999 Evaluation designs for adequacy, plausibility and probability of public health programme performance and impact. Int J Epidemiol 28: 10–18. 10195658 10.1093/ije/28.1.10

[6] Victora CG Habicht JP Bryce J, 2004 Evidence-based public health: moving beyond randomized trials. Am J Public Health 94: 400–405. 14998803 10.2105/ajph.94.3.400 PMC1448265

[7] Cornfield J, 1978 Randomization by group: a formal analysis. Am J Epidemiol 108: 100–102. 707470 10.1093/oxfordjournals.aje.a112592

[8] Kirkwood BR Cousens SN Victora CG de Zoysa I, 1997 Issues in the design and interpretation of studies to evaluate the impact of community-based interventions. Trop Med Int Health 2: 1022–1029. 9391504 10.1046/j.1365-3156.1997.d01-188.x

[9] Victora CG Black RE Boerma JT Bryce J, 2011 Measuring impact in the Millennium Development Goal era and beyond: a new approach to large-scale effectiveness evaluations. Lancet 377: 85–95. 20619886 10.1016/S0140-6736(10)60810-0

[10] Rowe AK, et al., 2007 Viewpoint: evaluating the impact of malaria control efforts on mortality in sub-Saharan Africa. Trop Med Int Health 12: 1524–1539. 18076561 10.1111/j.1365-3156.2007.01961.x

[11] de Savigny D Binka F, 2004 Monitoring future impact on malaria burden in sub-saharan Africa. Am J Trop Med Hyg 71: 224–231. 15331841

[12] Gething PW Noor AM Goodman CA Gikandi PW Hay SI Sharif SK Atkinson PM Snow RW, 2007 Information for decision making from imperfect national data: tracking major changes in health care use in Kenya using geostatistics. BMC Med 5: 37. 18072976 10.1186/1741-7015-5-37 PMC2225405

[13] Lim SS Fullman N Stokes A Ravishankar N Masiye F Murray CJ Gakidou E, 2011 Net benefits: a multicountry analysis of observational data examining associations between insecticide-treated mosquito nets and health outcomes. PLoS Med 8: e1001091. 21909249 10.1371/journal.pmed.1001091 PMC3167799

[14] Giardina F Kasasa S Sie A Utzinger J Tanner M Vounatsou P, 2014 Effects of vector-control interventions on changes in risk of malaria parasitaemia in sub-Saharan Africa: a spatial and temporal analysis. Lancet Glob Health 2: e601–e615. 25304636 10.1016/S2214-109X(14)70300-6

[15] Bhatt S, et al., 2015. The effect of malaria control on *Plasmodium falciparum* in Africa between 2000 and 2015. Nature 526: 207–211. 26375008 10.1038/nature15535 PMC4820050

[16] Wagenaar BH Sherr K Fernandes Q Wagenaar AC, 2016 Using routine health information systems for well-designed health evaluations in low- and middle-income countries. Health Policy Plan 31: 129–135. 25887561 10.1093/heapol/czv029 PMC4751224

[17] Kiberu VM Matovu JK Makumbi F Kyozira C Mukooyo E Wanyenze RK, 2014 Strengthening district-based health reporting through the district health management information software system: the Ugandan experience. BMC Med Inform Decis Mak 14: 40. 24886567 10.1186/1472-6947-14-40 PMC4030005

[18] MalERA Consultative Group on Monitoring Evaluation and Surveillance, 2011 A research agenda for malaria eradication: monitoring, evaluation, and surveillance. *PLoS Med 8:* e1000400. 10.1371/journal.pmed.1000400 PMC3026689 21311581

[19] World Health Organization, 2015 *Global Technical Strategy for Malaria 2016–2030* Geneva, Switzerland: WHO. Available at: http://www.who.int/malaria/publications/atoz/9789241564991/en/. Accessed November 18, 2015.

[20] Amouzou A Kachaka W Banda B Chimzimu M Hill K Bryce J, 2013 Monitoring child survival in ‘real time’ using routine health facility records: results from Malawi. Trop Med Int Health 18: 1231–1239. 23906285 10.1111/tmi.12167 PMC3787785

[21] Lippeveld T Sauerborn R Bodart C, 2000 *Design and Implementation of Health Information Systems* Report No. 9789241561990. Geneva, Switzerland: World Health Organization. Available at: http://apps.who.int/iris/bitstream/10665/42289/1/9241561998.pdf. Accessed November 18, 2015.

[22] Yukich JO Butts J Miles M Berhane Y Nahusenay H Malone JL Dissanayake G Reithinger R Keating J, 2014 A description of malaria sentinel surveillance: a case study in Oromia Regional State, Ethiopia. Malar J 13: 88. 24618105 10.1186/1475-2875-13-88 PMC3995772

[23] Bennett A, Avanceña ALV, Wegbreit J, Cotter C, Roberts K, Gosling RD, 2014 *Background Paper: The Private Sector’s Role in Malaria Surveillance: Global Health Group, University of California, San Francisco (UCSF)* Available at: http://globalhealthsciences.ucsf.edu/sites/default/files/content/ghg/mei-private-sectors-role-in-malaria-surveillance.pdf. Accessed April 19, 2016.

[24] Bennett A, et al., 2014 A methodological framework for the improved use of routine health system data to evaluate national malaria control programs: evidence from Zambia. Popul Health Metr 12: 30. 25435815 10.1186/s12963-014-0030-0 PMC4247605

[25] Comfort AB, et al., 2014 Hospitalizations and costs incurred at the facility level after scale-up of malaria control: pre-post comparisons from two hospitals in Zambia. Am J Trop Med Hyg 90: 20–32. 24218409 10.4269/ajtmh.13-0019 PMC3886421

[26] Otten M, et al., 2009 Initial evidence of reduction of malaria cases and deaths in Rwanda and Ethiopia due to rapid scale-up of malaria prevention and treatment. Malar J 8: 14. 19144183 10.1186/1475-2875-8-14 PMC2653503

[27] Rowe AK Kachur SP Yoon SS Lynch M Slutsker L Steketee RW, 2009 Caution is required when using health facility-based data to evaluate the health impact of malaria control efforts in Africa. Malar J 8: 209. 19728880 10.1186/1475-2875-8-209 PMC2743707

[28] Bonell CP Hargreaves J Cousens S Ross D Hayes R Petticrew M Kirkwood BR, 2011 Alternatives to randomisation in the evaluation of public health interventions: design challenges and solutions. J Epidemiol Community Health 65: 582–587. 19213758 10.1136/jech.2008.082602

[29] Chanda E Coleman M Kleinschmidt I Hemingway J Hamainza B Masaninga F Chanda-Kapata P Baboo KS Durrheim DN Coleman M, 2012 Impact assessment of malaria vector control using routine surveillance data in Zambia: implications for monitoring and evaluation. Malar J 11: 437. 23273109 10.1186/1475-2875-11-437 PMC3543298

[30] Sarrassat S Senghor P Le Hesran JY, 2008 Trends in malaria morbidity following the introduction of artesunate plus amodiaquine combination in M’lomp village dispensary, south-western Senegal. Malar J 7: 215. 18950485 10.1186/1475-2875-7-215 PMC2584070

[31] Gertler PJ Martinez S Premand P Rawlings LB Vermeersch CMJ, 2011 *Impact Evaluation in Practice*. Washington, DC: The World Bank.

[32] Bryce J Gilroy K Jones G Hazel E Black RE Victora CG, 2010 The Accelerated Child Survival and Development programme in West Africa: a retrospective evaluation. Lancet 375: 572–582. 20071020 10.1016/S0140-6736(09)62060-2

[33] Maude RJ, et al., 2014. Spatial and temporal epidemiology of clinical malaria in Cambodia 2004–2013. *Malar J 13:* 385. 10.1186/1475-2875-13-385 PMC4531392 25266007

[34] Ceesay SJ, et al., 2008 Changes in malaria indices between 1999 and 2007 in The Gambia: a retrospective analysis. Lancet 372: 1545–1554. 18984187 10.1016/S0140-6736(08)61654-2 PMC2607025

[35] Dhimal M Ahrens B Kuch U, 2014 Malaria control in Nepal 1963–2012: challenges on the path towards elimination. Malar J 13: 241. 24957851 10.1186/1475-2875-13-241 PMC4078365

[36] Alba S Hetzel MW Nathan R Alexander M Lengeler C, 2011 Assessing the impact of malaria interventions on morbidity through a community-based surveillance system. Int J Epidemiol 40: 405–416. 21216742 10.1093/ije/dyq240

[37] Kamuliwo M, et al., 2013 The changing burden of malaria and association with vector control interventions in Zambia using district-level surveillance data, 2006–2011. Malar J 12: 437. 24289177 10.1186/1475-2875-12-437 PMC3881017

[38] Ngomane L de Jager C, 2012 Changes in malaria morbidity and mortality in Mpumalanga Province, South Africa (2001–2009): a retrospective study. Malar J 11: 19. 22239855 10.1186/1475-2875-11-19 PMC3292497

[39] Nyarango PM, et al., 2006 A steep decline of malaria morbidity and mortality trends in Eritrea between 2000 and 2004: the effect of combination of control methods. Malar J 5: 33. 16635265 10.1186/1475-2875-5-33 PMC1501031

[40] Okiro EA Hay SI Gikandi PW Sharif SK Noor AM Peshu N Marsh K Snow RW, 2007 The decline in paediatric malaria admissions on the coast of Kenya. Malar J 6: 151. 18005422 10.1186/1475-2875-6-151 PMC2194691

[41] Okiro EA Bitira D Mbabazi G Mpimbaza A Alegana VA Talisuna AO Snow RW, 2011 Increasing malaria hospital admissions in Uganda between 1999 and 2009. BMC Med 9: 37. 21486498 10.1186/1741-7015-9-37 PMC3096581

[42] Okiro EA Kazembe LN Kabaria CW Ligomeka J Noor AM Ali D Snow RW, 2013 Childhood malaria admission rates to four hospitals in Malawi between 2000 and 2010. PLoS One 8: e62214. 23638008 10.1371/journal.pone.0062214 PMC3637378

[43] Penfold RB Zhang F, 2013 Use of interrupted time series analysis in evaluating health care quality improvements. Acad Pediatr 13: S38–S44. 24268083 10.1016/j.acap.2013.08.002

[44] Wagner AK Soumerai SB Zhang F Ross-Degnan D, 2002 Segmented regression analysis of interrupted time series studies in medication use research. J Clin Pharm Ther 27: 299–309. 12174032 10.1046/j.1365-2710.2002.00430.x

[45] Shadish WR Cook TD Campbell DT, 2002 *Experimental and Quasi-Experimental Designs for Generalized Causal Inference*. Belmont, CA: Wadsworth Cengage Learning.

[46] Karema C, et al., 2012 Trends in malaria cases, hospital admissions and deaths following scale-up of anti-malarial interventions, 2000–2010, Rwanda. Malar J 11: 236. 22823945 10.1186/1475-2875-11-236 PMC3502144

[47] Aregawi MW, et al., 2011 Reductions in malaria and anaemia case and death burden at hospitals following scale-up of malaria control in Zanzibar, 1999–2008. Malar J 10: 46. 21332989 10.1186/1475-2875-10-46 PMC3050777

[48] Aregawi M, et al., 2014 Time series analysis of trends in malaria cases and deaths at hospitals and the effect of antimalarial interventions, 2001–2011, Ethiopia. PLoS One 9: e106359. 25406083 10.1371/journal.pone.0106359 PMC4236017

[49] Kigozi R, et al., 2012 Indoor residual spraying of insecticide and malaria morbidity in a high transmission intensity area of Uganda. PLoS One 7: e42857. 22880123 10.1371/journal.pone.0042857 PMC3412792

[50] Landoh ED Tchamdja P Saka B Tint KS Gitta SN Wasswa P, Christiaan de J, 2012 Morbidity and mortality due to malaria in Est Mono district, Togo, from 2005 to 2010: a times series analysis. Malar J 11: 389. 23173765 10.1186/1475-2875-11-389 PMC3519571

[51] Bukirwa H Yau V Kigozi R Filler S Quick L Lugemwa M Dissanayake G Kamya M Wabwire-Mangen F Dorsey G, 2009 Assessing the impact of indoor residual spraying on malaria morbidity using a sentinel site surveillance system in western Uganda. Am J Trop Med Hyg 81: 611–614. 19815875 10.4269/ajtmh.2009.09-0126

[52] Santelli AC, et al., 2012 Effect of artesunate-mefloquine fixed-dose combination in malaria transmission in Amazon basin communities. Malar J 11: 286. 22905900 10.1186/1475-2875-11-286 PMC3472241

[53] Teklehaimanot HD Teklehaimanot A Kiszewski A Rampao HS Sachs JD, 2009 Malaria in São Tomé and Principe: on the brink of elimination after three years of effective antimalarial measures. Am J Trop Med Hyg 80: 133–140. 19141851

[54] Graves PM, et al., 2008 Effectiveness of malaria control during changing climate conditions in Eritrea, 1998–2003. Trop Med Int Health 13: 218–228. 18304268 10.1111/j.1365-3156.2007.01993.x

[55] Akin JS Hutchinson P, 1999 Health-care facility choice and the phenomenon of bypassing. Health Policy Plan 14: 135–151. 10538717 10.1093/heapol/14.2.135

[56] Schuurman N Fiedler RS Grzybowski SC Grund D, 2006 Defining rational hospital catchments for non-urban areas based on travel-time. Int J Health Geogr 5: 43. 17018146 10.1186/1476-072X-5-43 PMC1617091

[57] Alegana VA Wright JA Pentrina U Noor AM Snow RW Atkinson PM, 2012 Spatial modelling of healthcare utilisation for treatment of fever in Namibia. Int J Health Geogr 11: 6. 22336441 10.1186/1476-072X-11-6 PMC3292929

[58] Alexandrescu R O’Brien SJ Lyons RA Lecky FE Trauma A Research N, 2008 A proposed approach in defining population-based rates of major injury from a trauma registry dataset: delineation of hospital catchment areas (I). BMC Health Serv Res 8: 80. 18402693 10.1186/1472-6963-8-80 PMC2365946

[59] Huff DL, 1964 Defining and estimating a trading area. J Mark 28: 34–38.

[60] Luo J, 2014 Integrating the Huff Model and Floating Catchment Area Methods to analyze spatial access to healthcare services. T GIS 18: 436–448.

[61] Zinszer K Charland K Kigozi R Dorsey G Kamya MR Buckeridge DL, 2014 Determining health-care facility catchment areas in Uganda using data on malaria-related visits. Bull World Health Organ 92: 178–186. 24700977 10.2471/BLT.13.125260 PMC3949593

[62] Woolhouse ME, 1998 Patterns in parasite epidemiology: the peak shift. Parasitol Today 14: 428–434. 17040835 10.1016/s0169-4758(98)01318-0

[63] NASA Socioeconomic Data and Applications Center (SEDAC), 2016 *Gridded Population of the World, v4* Available at: http://sedac.ciesin.columbia.edu/data/collection/gpw-v4. Accessed August 25, 2016.

[64] Chandramohan D Jaffar S Greenwood B, 2002 Use of clinical algorithms for diagnosing malaria. Trop Med Int Health 7: 45–52. 11851954 10.1046/j.1365-3156.2002.00827.x

[65] Rowe AK de Savigny D Lanata CF Victora CG, 2005 How can we achieve and maintain high-quality performance of health workers in low-resource settings? Lancet 366: 1026–1035. 16168785 10.1016/S0140-6736(05)67028-6

[66] Larsen DA Bennett A Silumbe K Hamainza B Yukich JO Keating J Littrell M Miller JM Steketee RW Eisele TP, 2015 Population-wide malaria testing and treatment with rapid diagnostic tests and artemether-lumefantrine in southern Zambia: a community randomized step-wedge control trial design. Am J Trop Med Hyg 92: 913–921. 25802434 10.4269/ajtmh.14-0347 PMC4426577

[67] Willey BA Armstrong Schellenberg JR Maokola W Shirima K Chemba M Mshinda H Alonso P Tanner M Schellenberg D, 2011 Evaluating the effectiveness of IPTi on malaria using routine health information from sentinel health centres in southern Tanzania. Malar J 10: 41. 21320346 10.1186/1475-2875-10-41 PMC3055223

[68] German RR Lee LM Horan JM Milstein RL Pertowski CA Waller MN; Guidelines Working Group Centers for Disease Control and Prevention, 2001 Updated guidelines for evaluating public health surveillance systems: recommendations from the Guidelines Working Group. MMWR Recomm Rep 50: 1–35. 18634202

[69] Okiro EA Alegana VA Noor AM Mutheu JJ Juma E Snow RW, 2009 Malaria paediatric hospitalization between 1999 and 2008 across Kenya. BMC Med 7: 75. 20003178 10.1186/1741-7015-7-75 PMC2802588

[70] Gething PW Noor AM Gikandi PW Hay SI Nixon MS Snow RW Atkinson PM, 2008 Developing geostatistical space-time models to predict outpatient treatment burdens from incomplete national data. Geogr Anal 40: 167–188. 19325928 10.1111/j.1538-4632.2008.00718.x PMC2660576

[71] Cibulskis RE Aregawi M Williams R Otten M Dye C, 2011 Worldwide incidence of malaria in 2009: estimates, time trends, and a critique of methods. PLoS Med 8: e1001142. 22205883 10.1371/journal.pmed.1001142 PMC3243721

[72] Mubiru D, et al., 2015 Evaluation of integrated community case management in eight districts of central Uganda. PLoS One 10: e0134767. 26267141 10.1371/journal.pone.0134767 PMC4534192

[73] Svoronos T Mate KS, 2011 Evaluating large-scale health programmes at a district level in resource-limited countries. Bull World Health Organ 89: 831–837. 22084529 10.2471/BLT.11.088138 PMC3209726

[74] Masanja H, et al., 2008 Child survival gains in Tanzania: analysis of data from demographic and health surveys. Lancet 371: 1276–1283. 18406862 10.1016/S0140-6736(08)60562-0

[75] Last JM, 2001. *A Dictionary of Epidemiology*. 4th ed. New York: Oxford University Press.

[76] Hill AB, 1965 The environment and disease: association or causation? Proc R Soc Med 58: 295–300. 14283879 10.1177/003591576505800503 PMC1898525

[77] Hofler M, 2005 The Bradford Hill considerations on causality: a counterfactual perspective. Emerg Themes Epidemiol 2: 11. 16269083 10.1186/1742-7622-2-11 PMC1291382

[78] Rowe AK Onikpo F Lama M Osterholt DM Deming MS, 2011 Impact of a malaria-control project in Benin that included the integrated management of childhood illness strategy. Am J Public Health 101: 2333–2341. 21566036 10.2105/AJPH.2010.300068 PMC3222441

[79] Bryce J Victora CG Habicht JP Vaughan JP Black RE, 2004 The multi-country evaluation of the integrated management of childhood illness strategy: lessons for the evaluation of public health interventions. Am J Public Health 94: 406–415. 14998804 10.2105/ajph.94.3.406 PMC1448266

[80] Ye Y, et al., 2017 Framework for evaluating the health impact of the scale-up of malaria control interventions on all-cause child mortality in sub-Saharan Africa. Am J Trop Med Hyg 97 (Suppl 3): 9–19. 28990923 10.4269/ajtmh.15-0363 PMC5619929

[81] Bhattarai A, et al., 2007 Impact of artemisinin-based combination therapy and insecticide-treated nets on malaria burden in Zanzibar. PLoS Med 4: e309. 17988171 10.1371/journal.pmed.0040309 PMC2062481

[82] Johansson EW Gething PW Hildenwall H Mappin B Petzold M Peterson SS Selling KE, 2015 Effect of diagnostic testing on medicines used by febrile children less than five years in 12 malaria-endemic African countries: a mixed-methods study. Malar J 14: 194. 25957881 10.1186/s12936-015-0709-0 PMC4432948

[83] Snow RW Kibuchi E Karuri SW Sang G Gitonga CW Mwandawiro C Bejon P Noor AM, 2015 Changing malaria prevalence on the Kenyan Coast since 1974: climate, drugs and vector control. PLoS One 10: e0128792. 26107772 10.1371/journal.pone.0128792 PMC4479373

[84] Pindolia DK Garcia AJ Wesolowski A Smith DL Buckee CO Noor AM Snow RW Tatem AJ, 2012 Human movement data for malaria control and elimination strategic planning. Malar J 11: 205. 22703541 10.1186/1475-2875-11-205 PMC3464668

[85] Tatem AJ, et al., 2014 Integrating rapid risk mapping and mobile phone call record data for strategic malaria elimination planning. Malar J 13: 52. 24512144 10.1186/1475-2875-13-52 PMC3927223

[86] Hay SI Omumbo JA Craig MH Snow RW, 2000 Earth observation, geographic information systems and *Plasmodium falciparum* malaria in sub-Saharan Africa. Adv Parasitol 47: 173–215. 10997207 10.1016/s0065-308x(00)47009-0 PMC3164801

[87] Weiss DJ Mappin B Dalrymple U Bhatt S Cameron E Hay SI Gething PW, 2015 Re-examining environmental correlates of *Plasmodium falciparum* malaria endemicity: a data-intensive variable selection approach. Malar J 14: 68. 25890035 10.1186/s12936-015-0574-x PMC4333887

[88] Gething PW Patil AP Smith DL Guerra CA Elyazar IRF Johnston GL Tatem AJ Hay SI, 2011 A new world malaria map: *Plasmodium falciparum* endemicity in 2010. Malar J 10: 378. 22185615 10.1186/1475-2875-10-378 PMC3274487

[89] Malaria Atlas Project, 2016 *Malaria Endemicity Maps* Available at: http://www.map.ox.ac.uk/browse-resources/?region=&country=&topic=endemicity&subtopic=. Accessed April 19, 2016.

[90] Moyes CL Temperley WH Henry AJ Burgert CR Hay SI, 2013 Providing open access data online to advance malaria research and control. Malar J 12: 161. 23680401 10.1186/1475-2875-12-161 PMC3662599

[91] Larsen DA Hutchinson P Bennett A Yukich J Anglewicz P Keating J Eisele TP, 2014 Community coverage with insecticide-treated mosquito nets and observed associations with all-cause child mortality and malaria parasite infections. Am J Trop Med Hyg 91: 950–958. 25200267 10.4269/ajtmh.14-0318 PMC4228892

[92] Eisele TP Larsen DA Anglewicz PA Keating J Yukich J Bennett A Hutchinson P Steketee RW, 2012 Malaria prevention in pregnancy, birthweight, and neonatal mortality: a meta-analysis of 32 national cross-sectional datasets in Africa. Lancet Infect Dis 12: 942–949. 22995852 10.1016/S1473-3099(12)70222-0

[93] Zeger SL Irizarry R Peng RD, 2006 On time series analysis of public health and biomedical data. Annu Rev Public Health 27: 57–79. 16533109 10.1146/annurev.publhealth.26.021304.144517

[94] Abeku TA de Vlas SJ Borsboom G Teklehaimanot A Kebede A Olana D van Oortmarssen GJ Habbema JDF, 2002 Forecasting malaria incidence from historical morbidity patterns in epidemic-prone areas of Ethiopia: a simple seasonal adjustment method performs best. Trop Med Int Health 7: 851–857. 12358620 10.1046/j.1365-3156.2002.00924.x

[95] Alegana VA Atkinson PM Wright JA Kamwi R Uusiku P Katokele S Snow RW Noor AM, 2013 Estimation of malaria incidence in northern Namibia in 2009 using Bayesian conditional-autoregressive spatial-temporal models. Spat Spatio-Temporal Epidemiol 7: 25–36. 10.1016/j.sste.2013.09.001 PMC3839406 24238079

[96] Louis VR, et al., 2015 An insecticide-treated bed-net campaign and childhood malaria in Burkina Faso. Bull World Health Organ 93: 750–758. 26549902 10.2471/BLT.14.147702 PMC4622154

[97] Maes P Harries AD Van den Bergh R Noor A Snow RW Tayler-Smith K Hinderaker SG Zachariah R Allan R, 2014 Can timely vector control interventions triggered by atypical environmental conditions prevent malaria epidemics? A case-study from Wajir County, Kenya. PLoS One 9: e92386. 24699034 10.1371/journal.pone.0092386 PMC3974701

[98] Masaninga F, et al., 2013 Review of the malaria epidemiology and trends in Zambia. Asian Pac J Trop Biomed 3: 89–94. 23593585 10.1016/S2221-1691(13)60030-1 PMC3627166

[99] Thang ND Erhart A, Hung le X, Thuan le K, Xa NX, Thanh NN, Ky PV, Coosemans M, Speybroeck N, D’Alessandro U, 2009 Rapid decrease of malaria morbidity following the introduction of community-based monitoring in a rural area of central Vietnam. Malar J 8: 3. 19123932 10.1186/1475-2875-8-3 PMC2657912

[100] Yapabandara MA Sarmento R de Fatima Mota Mdo R, don Bosco J, Martins N, Wickremasinghe AR, 2015 Evidence-based malaria control in Timor Leste from 2006 to 2012. Malar J 14: 109. 25890294 10.1186/s12936-015-0614-6 PMC4363051

[101] Konchom S Singhasivanon P Kaewkungwal J Chupraphawan S Thimasarn K Kidson C Rojanawatsirivet C Yimsamran S Looareesuwan S, 2003 Trend of malaria incidence in highly endemic provinces along the Thai borders, 1991–2001. Southeast Asian J Trop Med Public Health 34: 486–494. 15115117

[102] Mufunda J, et al., 2007 Roll back malaria—an African success story in Eritrea. S Afr Med J 97: 46–50. 17378282

[103] Mukonka VM, et al., 2014 High burden of malaria following scale-up of control interventions in Nchelenge District, Luapula Province, Zambia. Malar J 13: 153. 24755108 10.1186/1475-2875-13-153 PMC4016669

